# Complete Genome Sequence of the *Campylobacter coli* Clinical Isolate 15-537360

**DOI:** 10.1128/genomeA.01056-13

**Published:** 2013-12-12

**Authors:** Bruce M. Pearson, Assaf Rokney, Lisa C. Crossman, William G. Miller, John Wain, Arnoud H. M. van Vliet

**Affiliations:** Institute of Food Research, Gut Health and Food Safety Programme, Norwich Research Park, Norwich, United Kingdoma; Ministry of Health, Government Central Laboratories, Jerusalem, Israelb; The Genome Analysis Centre, Norwich Research Park, Norwich, United Kingdomc; University of East Anglia, School of Biological Sciences, Norwich Research Park, Norwich, United Kingdomd; U.S. Department of Agriculture, ARS, WRRC, Produce Safety and Microbiology, Albany, California, USAe; University of East Anglia, Norwich Medical School, Norwich Research Park, Norwich, United Kingdomf

## Abstract

*Campylobacter coli* strain 15-537360 was originally isolated in 2001 from a 42-year-old patient with gastroenteritis. Here, we report its complete genome sequence, which comprises a 1.7-Mbp chromosome and a 29-kbp conjugative cryptic plasmid. This is the first complete genome sequence of a clinical isolate of *C. coli*.

## GENOME ANNOUNCEMENT

The food-borne bacterial pathogens of the genus *Campylobacter* are the most common cause of bacterial infectious intestinal disease in the developed world, with an estimated annual incidence of ~400,000 cases in the United Kingdom (1). The majority of cases are caused by *Campylobacter jejuni*, but at least 10% of these cases are caused by *Campylobacter coli* (2). *C. coli* has a wider range of infective sources than *C. jejuni*, and the unique risk factors for *C. coli* include the consumption of game, tripe, and bottled water and recreational swimming (2, 3). However, despite the large number of cases associated with *C. coli*, relatively little is known about its transmission routes or mechanisms of pathogenicity.

Complete genome sequences of *C. jejuni* reference isolates have been published (4, 5), and currently, >2,000 genome sequences of *C. jejuni* and *C. coli* are available in public databases, such as GenBank and BIGSdb (6). However, the large majority of these genome sequences are not annotated and are in draft (incomplete) format. For *C. coli*, there were no finished genome sequences available until the recent release of the genome sequence of the gentamicin-resistant poultry isolate *C. coli* CVM N29710 (7), but complete genome sequences have not been released for clinical isolates of *C. coli*. Here, we announce the completion and annotation of the genome and plasmid sequences of the clinical *C. coli* isolate 15-537360. This clinical isolate was found from multilocus sequence typing (MLST) to be from sequence type 855 (ST-855) and belongs to clonal complex 828 (CC828) (8, 9).

Genomic DNA was subjected to whole-genome sequencing on the Illumina MiSeq platform (Illumina, Inc.), with read lengths of 150 nucleotides, using paired-end sequencing with a library insert size of ~500 nucleotides. The subsequent assembly yielded 16 contigs, which allowed for a reference-based assembly using *C. coli* RM2228 (5). A number of primers were designed to amplify and sequence all joins and to complete gap closure. A number of inversions that were compared to *C. jejuni* NCTC 11168 were tested by PCR methods to check that the genome was not misassembled. A plasmid was present as a single contig, and so the ends were checked for circularity by PCR. The whole genome of *C. coli* isolate 15-537360 is 1,658,751 bp in length (31.5% G+C content), whereas the circular plasmid is 26,269 bp (29.4% G+C content). The initial annotation was performed using RAST (10) and was manually inspected and corrected for translation start sites and potential pseudogenes. The genome contains 1,588 annotated open reading frames (ORFs), and an additional 38 genes are currently marked as pseudogenes. Comparison to the completed genome sequence of *C. coli* CVM N29710 (7) showed that these strains share 1,486 orthologs, with *C. coli* 15-537360 having 102 coding sequences (CDSs) not found in CVM N29710 and CVM N29710 having 143 CDSs that are absent in 15-537360. The plasmid has 33 CDSs and does not contain antibiotic resistance markers, but it does appear to be conjugative, with a type IV secretion system (11). The genome contains the previously described flagellar modification region (12) but lacks the fucose utilization cluster (13).

### Nucleotide sequence accession numbers.

The whole-genome sequence assembly and automatic annotation of *C. coli* 15-537360 and its plasmid have been deposited in EMBL/GenBank under accession no. CP006702 and CP006703, respectively.
